# Dosimetric validation and clinical implementation of two 3D dose verification systems for quality assurance in volumetric‐modulated arc therapy techniques

**DOI:** 10.1120/jacmp.v16i2.5190

**Published:** 2015-03-08

**Authors:** Francisco Clemente‐Gutiérrez, Consuelo Pérez‐Vara

**Affiliations:** ^1^ Sección de Radiofísica Servicio de Oncología Radioterápica, Hospital Central de la Defensa “Gómez Ulla” Madrid Spain

**Keywords:** VMAT QA, pretreatment verifications, Mobius3D, COMPASS

## Abstract

A pretreatment quality assurance program for volumetric techniques should include redundant calculations and measurement‐based verifications. The patient‐specific quality assurance process must be based in clinically relevant metrics. The aim of this study was to show the commission, clinical implementation, and comparison of two systems that allow performing a 3D redundant dose calculation. In addition, one of them is capable of reconstructing the dose on patient anatomy from measurements taken with a 2D ion chamber array. Both systems were compared in terms of reference calibration data (absolute dose, output factors, percentage depth‐dose curves, and profiles). Results were in good agreement for absolute dose values (discrepancies were below 0.5%) and output factors (mean differences were below 1%). Maximum mean discrepancies were located between 10 and 20 cm of depth for PDDs (‐2.7%) and in the penumbra region for profiles (mean DTA of 1.5 mm). Validation of the systems was performed by comparing point‐dose measurements with values obtained by the two systems for static, dynamic fields from AAPM TG‐119 report, and 12 real VMAT plans for different anatomical sites (differences better than 1.2%). Comparisons between measurements taken with a 2D ion chamber array and results obtained by both systems for real VMAT plans were also performed (mean global gamma passing rates better than 87.0% and 97.9% for the 2%/2 mm and 3%/3 mm criteria). Clinical implementation of the systems was evaluated by comparing dose‐volume parameters for all TG‐119 tests and real VMAT plans with TPS values (mean differences were below 1%). In addition, comparisons between dose distributions calculated by TPS and those extracted by the two systems for real VMAT plans were also performed (mean global gamma passing rates better than 86.0% and 93.0% for the 2%/2 mm and 3%/3 mm criteria). The clinical use of both systems was successfully evaluated.

PACS numbers: 87.56.Fc, 87.56.‐v, 87.55.dk, 87.55.Qr, 87.55.‐x, 07.57.Kp, 85.25.Pb

## I. INTRODUCTION

Intensity‐modulated radiation therapy (IMRT) treatments provide highly conformed dose distributions compared with traditional techniques.^(1)^ Improvements in the performance of multileaf collimator (MLC) systems, together with rotational capabilities recently implemented in conventional linacs, have made possible the development of volumetric‐modulated arc therapy (VMAT).^(2)^ Independent verification of the treatment planning system (TPS) calculations is an essential part of the quality assurance (QA) process in radiation therapy. This verification is traditionally based on manual monitor unit (MU) calculation methods for 3D conformal radiotherapy (3D CRT) treatments.^3^, ^4^, ^5^, ^6^, ^7^ The complexity present in the modulated treatments requires an introduction of a comprehensive quality assurance program aimed at its implementation.^(8)^ Such QA routines must take into account two approaches. On the one hand, an independent verification of the TPS dose calculations should be carried out. One way to fulfill this requirement consists of the application of Monte Carlo calculations for the independent verification of the treatment plan.^(9)^ The main limitation in the application of these techniques is the calculation time. Other solutions are based on simpler algorithms,^10^ like modified Clarkson methods^(11)^ and extensions with the inclusion of head scatter.^(12)^ In addition, the pretreatment QA measurement‐based process must be considered to ensure the correct information flow from TPS plan calculation to treatment delivery in the linac by means of the record and verify system (R&V). The usual method to perform this QA consists of comparing dose distribution measurements acquired with phantoms/detectors of regular geometries with TPS calculations made under the same conditions.^13^, ^14^, ^15^ Volumetric treatments have incorporated specifically developed solutions for these techniques.^16^, ^17^ Dose distribution comparisons tend to involve gamma index‐based analyses.^(18)^ Several studies have shown tolerances and action levels in the IMRT treatment verifications^14^, ^19^, ^20^, ^21^, ^22^ by means of the previous methods.

The current clinical research related to the verification and QA in IMRT treatment delivery, however, has introduced a fundamental issue. Commercial solutions for redundant verifications in modulated treatments have usually assumed simple situations, like homogeneous geometries or single‐point calculations,^14^, ^23^, ^24^ which are results with no clinical relevance. Likewise, the results derived from the usual individualized pretreatment QA tools have not been related with clinically relevant dosimetric errors on patient dose delivery.^25^, ^26^ The results of the measurements and analyses performed in pretreatment IMRT QA must be suitably correlated with implications of possible mistakes during TPS calculations and real treatment delivery on the basis of new clinically relevant metrics. The background to set up these metrics must be the patient dose estimation from typical QA measurements. If the reconstructed dose on patient CT could be performed from measurements, then clinically relevant parameters, such as dose‐volume histograms (DVH), could be extracted. In addition, redundant calculations must be considered in the same scope.^(25)^ Recently, new systems that allow setting the acceptance criteria for modulated treatments from DVH‐based metrics have been introduced.^(27)^ These solutions are further necessary in VMAT QA, where the synchronization of all variable parameters raises the complexity in treatment delivery from traditional IMRT techniques. Two‐dimensional (2D) ion chamber arrays, together with the suitable accessories, are adequate tools to extract as much information as possible from dynamic treatments.^(28)^ This paper shows the commissioning, comparison, and clinical implementation of two systems that allow performing 3D redundant dose calculations for VMAT secondary verifications. In addition, the second one is capable of reconstructing the dose on patient anatomy from measurements taken with 2D ion chamber arrays.

## II. MATERIALS AND METHODS

### A. Treatment unit and TPS

VMAT treatments were delivered in our institution with a 6 MV Synergy (Elekta, Stockholm, Sweden) machine. Plans were generated with Monaco 3.1 (Elekta).

### B. Mobius3D system description

Mobius3D software (Mobius Medical Systems, Houston, TX) provides an independent dose calculation engine aimed at the verification of treatments generated by TPS. DICOM treatment plan data (CT images, RT Plan, RT Struct and RT Dose) are needed as initial information. Mobius3D utilizes stock reference values for common linear accelerators to model beams. Users can choose these average models or fit usual parameters, such as percentage depth dose curves (PDDs), output factors (OFs), and off‐axis ratios (OARs), to scale the model correctly. In order to model the fluence, the system starts from a uniform map, adding layers of specific features for each linac (for instance, MLC characteristics and transmission or flattening filter properties). The software uses a collapsed cone convolution/superposition algorithm^29^, ^30^, ^31^ independently developed and updated from its original conception.^32^, ^33^, ^34^, ^35^ The algorithm is accelerated through graphic processing units (GPUs). A set of 144 isotropically spaced cones are used for each calculation point. Point dose kernels have been obtained with some refinements,^36^, ^37^ compared with the original study by Mackie et al.^(30)^ GPU‐based calculations increase the calculation speed significantly compared with CPUs.

### C. COMPASS system description

COMPASS (v. 3.0) (IBA Dosimetry, Schwarzenbruck, Germany) consists of two different elements: a detector device and calculation, reconstruction, and analysis software. The underlying idea is to reconstruct the dose on patient CT from measurements taken with the associated detector. In addition, it provides an independent dose calculation engine that ensures a redundant verification of TPS treatment, as the Mobius3D system. Below, a detailed description of each element is performed.

#### C.1 Detector device

**Figure 1:**
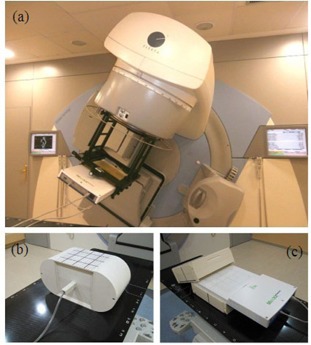
Phantoms used for measurements: (a) MatriXX array inserted in a holder attached to the gantry, (b) EasyCube phantom for point dose measurements, and (c) MatriXX array inserted in MultiCube phantom.

The detector system is a 2D ion chamber array (${\text{MatriXX}}^{\text{Evolution}}$, IBA Dosimetry). It consists of 1020 ion chambers with $0.08\;{\text{cm}}^{3}$ that covers an active area of $24.4\times 24.4\;{\text{cm}}^{2}$ (the distance between them is 7.619 mm). The versatility of the device is well known both for QA of treatment units and IMRT and VMAT verification.^38^, ^39^ As the detector element in the COMPASS system, it must be attached to the treatment unit head with a holder in order to ensure a rigid rotation of the device with the gantry. A buildup layer of 2.5 cm can be placed on the device, into the holder. With this arrangement (Fig. 1), the source‐to‐detector distance is 100 cm. The dose reconstruction process requires associating the measured fluence to its detection angle. An angle sensor is attached to the gantry, collecting angular information of each measurement. The sensor has a ±0.6° angular tolerance and must be calibrated previously by the user.

#### C.2 Calculation, reconstruction, and analysis software

Both features offered by COMPASS (dose calculation and reconstruction from measurements) rise from the beam modeling process, fitting basic parameters: photon and electron spectra, beam quality variation, source parameters or tongue and groove. Therefore, usual TPS commissioning measurements are required: relative distributions (profiles and PDDs), OFs, and absolute dose values. The model connects with a collapsed cone^29^, ^31^ convolution/superposition algorithm that calculates the dose on the patient CT.

A commissioning process is required for the MatriXX device. Initially, both background (20 s) and preirradiation measurements (5 Gy or higher) must be performed. A square field acquisition ($10\times 10\;{\text{cm}}^{2}$) is needed in order to check and correct detector shifting and rotation. A measurement with a known dose reference field must be performed to establish the absolute dose calibration. Sampling time was 250 ms for all measurements.

### D. Validation of the systems

#### D.1 Comparison with reference calibration

Square fields (2×2, 3×3, 4×4, 5×5, 7×7, 10×10, and $20\times 20\;{\text{cm}}^{2}$) were calculated on a homogeneous water phantom by the two systems. The source‐to‐surface distance (SSD) was 100 cm. Results were compared with reference calibration measurements taken with a water tank (${\text{Blue Phantom}}^{2}$, IBA Dosimetry). Absolute dose was compared in terms of calibration factor for the Synergy beam. The reference conditions were a field size of $10\times 10\;{\text{cm}}^{2}$ and depth of maximum. In addition, OFs were extracted and compared for previous fields. Measurements were performed with both Farmer FC65 and CC04 ion chambers (Scanditronix‐Wellhöfer/ IBA Dosimetry America, Inc., Bartlett, TN). The CC04 chamber was used in measurements for fields smaller than $4\times 4\;{\text{cm}}^{2}$. Chamber reading conversion to dose was performed by means of usual protocols.^(40)^

Relative dose distributions (PDDs and profiles at depth of maximum) were evaluated by computing mean differences and by means of a one‐dimensional gamma analysis with two criteria (2%/2 mm and 3%/3 mm, local normalization with no low‐dose threshold) in comparisons with water tank measurements. Relative measurements were acquired with a CC13 ion chamber (Scanditronix‐Wellhöfer). Depth dose curves were divided into four regions: buildup, maximum, depths between maximum and 10 cm, and depths between 10 cm and 20 cm. Profile curves were divided into three regions — outside the treatment field, penumbra, and inside the treatment field — for both in‐plane and cross‐plane sections. Penumbra regions, with steep dose gradients, were analyzed using distance‐to‐agreement (DTA) tests, rather than dose differences. An in‐house developed software was used to perform previous gamma and DTA tests.

#### D.2 Validation tests with static square fields and dynamic TG‐119 test plans

Tests with static regular fields were performed. Simple plans (square $10\times 10\;{\text{cm}}^{2}$ and 100 MU fields) were generated with single (anterior) and multiple fields (lateral opposed and four field box). In addition, following the guidelines of AAPM Task Group 119,^13^, ^21^ VMAT test plans were computed by the TPS with constraints defined in the report for PTVs and organs at risk (ORs) applied for each structure set. Dose calculation capabilities for both systems were assessed in more complex cases. Previous static fields and VMAT plans were delivered and calculated by both systems on a homogeneous phantom, comparing point dose calculations with ion chamber measurements. A phantom commonly involved in IMRT and VMAT verifications (EasyCube, IBA Dosimetry) (Fig. 1) was used. Measurements were performed with a CC04 ion chamber. The conversion factor from charge to dose using this phantom was extracted by comparing measurements taken under reference conditions in water with those performed in plastic. The isocenter of the test plans was matched with the phantom center. The measurement point was selected inside the PTV in all cases.

#### D.3 Validation with real VMAT patient plans

In order to evaluate different types of PTVs and locations, VMAT plans for four anatomical sites were generated with the TPS: head and neck (two treatments), thoracic (two lung treatments), abdominal (two gastric treatments) and pelvic (six prostate treatments, taking two from each usual staging: high‐, intermediate‐, and low‐risk). Representative point‐dose values obtained by the two systems were compared with ion chamber measurements (CC04) performed on the EasyCube phantom with the same arrangement. Likewise, planar dose distributions measured with MatriXX were compared with those generated by Mobius3D and COMPASS, with the same experimental setup, by means of gamma analysis (2%/2 mm and 3%/3 mm, global normalization to maximum with a low‐dose threshold at 10% of global maximum). It consisted of the detector array inserted in a homogeneous cubic phantom (MultiCube) (Fig. 1). The phantom was stationary on the linac couch while treatment was dynamically delivered on it. The thickness of both the anterior and backscatter buildup layers was 11 cm. Detector setup robustness allows different arrangements in order to perform coronal and sagittal measurements. MatriXX dose measurements were dependent on the angle of the beam. Angular correction factors must be incorporated to solve this dependency.^(39)^ Angular information could be collected with the COMPASS angle sensor previously described. OmniPro I'mRT (IBA Dosimetry America, Inc.) was used as analysis software.

### E. Clinical implementation of the systems

#### E.1 Clinical implementation tests with static square fields and dynamic TG‐119 test plans

Previous static regular fields were calculated with TPS over TG‐119 test cases in order to test DVH comparison modules. The dose received by TG‐119 test plan structures was determined by the two systems for the previously described regular and VMAT plans. Relevant dosimetric parameters, according to the TG‐119 report, were extracted and compared with TPS values for each test and structure.

#### E.2 Clinical implementation with real VMAT patient plans

Previous VMAT plans for each anatomical site were compared with TPS values using the clinical metrics previously defined. The process was carried out by evaluating representative dosimetric parameters from DVHs. ICRU recommendations for recording and reporting IMRT treatments (ICRU Report 83)^(41)^ were used to extract evaluation parameters for PTVs ($D_{98},D_{2},D_{50}$, $D_{\text{mean}}$). Maximum and mean doses were obtained for ORs. In addition, classical^(42)^ and recently reviewed dose constraints (QUANTEC)^(43)^ were reported for normal tissue. Comparisons with TPS by means of global gamma passing rates for all structures were reported with two criteria (2%/2 mm and 3%/3 mm, global normalization to maximum, with a low‐dose threshold at 10% of global maximum). The COMPASS system is capable of reporting local gamma 3D analysis, in contrast to Mobius3D. Local 2%/2 mm gamma passing rates were also reported for COMPASS dose calculation and reconstruction.

### F. Remarks about TPS dose calculation for plan verification

Collapsed cone algorithms of Mobius3D and COMPASS are based in dose engines that perform and report calculations in terms of the absorbed dose to water ($D_{w}$). In order to take into account patient heterogeneities properly, media are considered as water with different electronic densities. All TPS calculations presented in this study were performed using Monaco 3.1 (Elekta), with a Monte Carlo calculation algorithm, working in terms of the absorbed dose to medium ($D_{m}$). However, clinical implementation of Monte Carlo algorithms can lead to significant discrepancies between $D_{w}$ and $D_{m}$.^44^, ^45^, ^46^ In the AAPM report of the Task Group 105,^(45)^ recommendations about the conversion of $D_{m}$ to $D_{w}$ and its reporting have been described. Previous discussion led to performing the DVH‐based comparisons with the same criterion ($D_{w}$). All plans described in the present study were initially planned in terms of $D_{m}$ and recalculated in terms of $D_{w}$. (Monaco has implemented the two features.)

### G. Statistical analysis

Results were described as mean ± standard deviation (SD). Data were compared using a paired and two‐tailed Student's *t*‐test. The difference was considered statistically significant for p‐values<0.05.

## III. RESULTS

### A. Validation of the systems

#### A.1 Comparison with reference calibration

Absolute dose values extracted from both systems (Mobius3D [M3D], COMPASS dose calculation [CC], and COMPASS dose reconstruction [CR]) are shown in Table 1. Discrepancies were below 0.5%.

OFs are shown in Table 1. Maximum discrepancy was −3.4% for the $2\times 2\;{\text{cm}}^{2}$ COMPASS reconstructed field. Mean differences for M3D, CC, and CR were −0.8%±1.2%, −0.3%±0.3%, and −0.9%±1.4%, respectively.

Mean differences and gamma passing rates obtained with two different criteria for PDDs are shown in Table 2. Differences increased with depth, and they were statistically significant for comparisons between M3D and COMPASS in the region between the maximum and a depth of 20 cm (maximum mean difference of −2.7%±0.2% for M3D). Passing rates were better for COMPASS than those from M3D for both gamma criteria (p‐values of 0.03 and 0.02 for both CC and CR and for the 2%/2 mm and 3%/3 mm criteria, respectively).

Profile comparisons are shown in Table 3, computing mean differences (for outside and inside the field regions), mean DTA values (for penumbra regions), and gamma passing rates with two different criteria. Maximum discrepancies for in‐plane and cross‐plane sections were found outside (mean value of 1.4%±1.8%) and inside the treatment field (mean value of −0.9%±2.2%) for the M3D results. Maximum DTA values were found for M3D for both in‐plane (mean value of 0.9 mm±0.6 mm for $3\times 3\;{\text{cm}}^{2}$ field) and cross‐plane (1.5 mm±0.2 mm for $20\times 20\;{\text{cm}}^{2}$ field) sections. For in‐plane profiles, COMPASS results were better than M3D results outside the beam region (p<0.01 for both CC and CR comparisons). In addition, CC results were better than CR results outside and inside the field (p=0.01 and p=0.02). In the penumbra region of the in‐plane sections, COMPASS results were better than M3D results (p<0.01 for CC and p=0.01 for CR comparisons). For cross‐plane profiles, CC results were better than M3D results outside the beam (p=0.03). Furthermore, CC results were better than CR results in the beam region (p=0.01). In the penumbra region of the cross‐plane sections, COMPASS results were also better than M3D results (p=0.01 for both CC and CR comparisons). The remaining differences were not statistically significant, including gamma passing rates for profile comparisons.

**Table 1:** Comparisons of absolute dose values and output factors (OFs) for Mobius3D and COMPASS dose calculation and reconstruction

*Reference values*		*Mobius3D*		*COMPASS*			
		*Calculation*	*Diff (%)*	*Calculation*	*Diff (%)*	*Reconstruction*	*Diff (%)*
CF	0.685	0.685	−0.0	0.682	−0.4	0.685	0.0
Field	OF						
2	0.816	0.791	−3.1	0.811	−0.6	0.788	−3.4
3	0.856	0.844	−1.4	0.852	−0.5	0.842	−1.6
4	0.884	0.882	−0.2	0.880	−0.5	0.874	−1.1
5	0.908	0.901	−0.7	0.906	−0.2	0.902	−0.6
7	0.950	0.948	−0.2	0.952	0.2	0.949	−0.1
10	1.000	1.000	0.0	1.000	0.0	1.000	0.0
20	1.096	1.098	0.2	1.094	−0.2	1.105	0.8

CF=calibration factor; OF=output factor.

**Table 2:** Comparisons for percentage depth‐dose curves (PDDs) with reference measurements taken with water tank, for Mobius3D, Compass dose calculation, and reconstruction. PDDs were divided into four regions (buildup, maximum, maximum −10 cm depth, 10 cm−20 cm depth). The curves were compared by means of mean differences and gamma passing rates (2%/2 mm and 3%/3 mm, local normalization with no low‐dose threshold)

*Field Size*	*Mean Differences (%)*				*Gamma Passing Rates (%)*	
	*Buildup*	*Maximum*	*Maximum – 10 cm*	*10 cm–20 cm*	*Gamma* 2%/2 mm	*Gamma* 3%/3 mm
*Mobius3D*						
2	0.6±1.3	−0.1	−1.9±0.9	−3.2±0.2	44.6	98.5
3	0.8±0.8	−0.1	−1.8±0.9	−3.1±0.2	44.8	98.8
4	0.9±1.0	0.0	−2.1±1.0	−3.1±0.1	38.7	98.8
5	0.8±1.0	0.0	−1.2±0.6	−2.0±0.2	98.8	99.6
7	−0.4±1.5	0.5	−1.0±0.6	−2.1±0.3	98.6	99.3
10	1.8±1.6	0.1	−1.9±0.7	−3.0±0.3	54.3	98.8
20	0.5±1.8	−0.9	−2.3±0.4	−2.1±0.2	96.8	99.4
*COMPASS Calculation*						
2	1.3±1.5	0.0	−1.0±0.5	−1.6±0.2	99.6	99.6
3	1.5±1.3	0.2	−0.9±0.5	−1.6±0.2	99.6	99.6
4	0.7±1.6	0.0	−1.1±0.4	−1.7±0.1	99.6	99.6
5	0.8±1.5	0.0	−0.4±0.2	−0.6±0.1	99.6	99.7
7	−0.1±0.8	−0.1	−0.1±0.2	−0.7±0.3	99.2	99.2
10	0.3±1.0	0.0	−0.8±0.4	−1.8±0.2	99.2	99.7
20	0.2±0.7	0.4	−0.6±0.3	−1.3±0.3	99.7	99.8
*COMPASS Reconstruction*						
2	1.3±1.5	0.0	−1.0±0.5	−1.6±0.2	99.6	99.6
3	1.2±1.3	0.2	−0.9±0.4	−1.7±0.2	99.6	99.6
4	0.7±1.6	0.0	−1.2±0.5	−1.8±0.1	99.6	99.6
5	0.8±1.5	0.0	−0.4±0.2	−0.6±0.1	99.6	99.7
7	−0.1±0.8	−0.1	−0.1±0.2	−0.7±0.3	99.1	99.2
10	0.4±1.0	0.0	−0.8±0.4	−1.7±0.3	99.2	99.7
20	0.2±0.6	0.4	−0.6±0.3	−1.3±0.3	99.7	99.8

**Table 3:** Comparisons for profiles with reference measurements taken with water tank, for Mobius3D, Compass dose calculation, and reconstruction. Profiles were divided into three regions (outside the field, penumbra, and inside the field) for both in‐plane and cross‐plane sections. Mean differences were extracted for the regions outside and inside the field. Mean distance‐to‐agreement (DTA) values were extracted for penumbra regions. In addition, gamma passing rates (local normalization with no low‐dose threshold) were extracted with two different criteria (2%/2 mm and 3%/3 mm)

	*Mean Differences (%)*			*Mean DTA (mm)*	*Gamma Passing Rates (%)*	
	*Field size*	*Out field*	*In field*	*Penumbra*	*Gamma* 2%/2 mm	*Gamma* 3%/3 mm
*Mobius3D*						
In‐plane	2	1.0±2.6	−2.8±3.3	0.7±0.4	100.0	100.0
	3	1.8±2.5	−2.4±3.7	0.9±0.6	94.2	100.0
	4	1.8±2.3	−1.4±2.9	0.9±0.5	94.7	100.0
	5	1.6±1.6	−1.5±3.8	0.7±0.4	98.1	100.0
	7	0.7±1.1	−0.3±3.6	0.7±0.4	100.0	100.0
	10	1.6±0.3	−0.4±2.1	0.6±0.4	100.0	100.0
	20	1.5±0.6	−0.1±1.7	0.8±0.5	99.8	100.0
Cross‐plane	2	0.1±0.7	−1.9±2.0	0.4±0.3	100.0	100.0
	3	0.7±0.5	−2.4±3.7	0.5±0.4	100.0	100.0
	4	0.9±1.1	−1.5±2.6	0.8±0.5	94.7	100.0
	5	1.1±0.7	−0.6±1.8	0.5±0.4	100.0	100.0
	7	0.3±0.8	−1.0±2.2	0.8±0.6	100.0	100.0
	10	0.8±0.9	0.3±1.2	1.1±0.5	100.0	100.0
	20	0.2±1.3	0.5±1.4	1.5±0.2	100.0	100.0
*COMPASS Calculation*						
In‐plane	2	0.1±1.2	−1.0±1.1	0.3±0.2	100.0	100.0
	3	0.2±0.8	−0.4±1.6	0.4±0.3	100.0	100.0
	4	0.3±1.5	−0.4±0.5	0.4±0.2	100.0	100.0
	5	−0.1±0.3	−0.5±0.8	0.2±0.1	100.0	100.0
	7	0.0±0.2	−0.2±0.3	0.1±0.1	100.0	100.0
	10	−0.5±1.4	−0.3±0.4	0.5±0.3	100.0	100.0
	20	−0.6±1.0	−0.6±1.0	0.5±0.2	100.0	100.0
Cross‐plane	2	0.2±1.0	−0.8±1.5	0.3±0.2	100.0	100.0
	3	0.4±0.6	−0.8±1.2	0.3±0.2	100.0	100.0
	4	0.5±0.9	−0.5±0.9	0.3±0.1	94.8	100.0
	5	0.6±0.8	−0.5±0.8	0.3±0.2	100.0	100.0
	7	0.2±0.4	−1.1±1.3	0.3±0.3	100.0	100.0
	10	−0.1±0.9	0.4±0.6	0.2±0.1	100.0	100.0
	20	−0.5±0.9	−0.2±0.6	0.3±0.2	100.0	100.0
*COMPASS Reconstruction*						
In‐plane	2	0.2±0.8	−1.8±2.1	0.3±0.2	100.0	100.0
	3	0.5±1.2	−1.4±1.7	0.4±0.3	100.0	100.0
	4	0.3±0.6	−1.8±3.2	0.3±0.3	98.2	100.0
	5	−0.1±0.3	−1.1±1.3	0.2±0.2	100.0	100.0
	7	0.2±0.4	−0.9±1.5	0.2±0.1	100.0	100.0
	10	−0.2±1.6	−0.2±1.2	0.8±0.3	100.0	100.0
	20	−0.2±1.4	−0.6±0.9	0.6±0.2	100.0	100.0
Cross‐plane	2	0.4±1.1	−1.3±1.9	0.3±0.2	100.0	100.0
	3	0.6±0.9	−1.2±1.7	0.3±0.2	100.0	100.0
	4	0.6±0.8	−1.1±1.7	0.3±0.2	94.7	100.0
	5	0.6±0.9	−0.8±1.1	0.2±0.1	100.0	100.0
	7	0.2±0.3	−1.7±2.1	0.5±0.3	100.0	100.0
	10	0.1±0.9	0.2±0.3	0.2±0.2	100.0	100.0
	20	0.2±1.1	−0.1±0.3	0.4±0.2	100.0	100.0

#### A.2 Validation tests with static square fields and dynamic TG‐119 test plans

**Figure 2:**
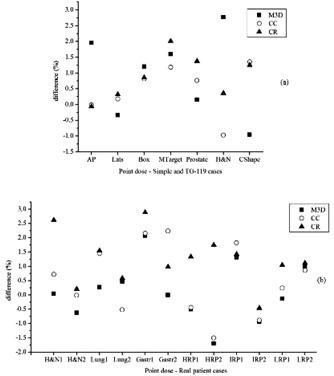
Relative differences between ion chamber measurements and dose extracted by the two systems: Mobius3D (M3D), COMPASS calculation (CC), and reconstruction (CR). Values were plotted for (a) static (anterior [AP], laterals [Lats], 4 field box [Box]), and dynamic TG‐119 plans (MultiTarget [MTarget], mock prostate [Prostate], mock head and neck [H&N], and CShape target [CShape]); and for (b) 12 real plans: 2 head and neck (H&N), 2 lung, 2 gastric (Gastr), and 6 pelvic (2 high‐ [HRP], 2 intermediate‐ [IRP] and 2 low‐risk prostate [LRP]) treatments.

Differences between point‐dose values obtained by both systems and measured with ion chamber are shown in Fig. 2. Mean discrepancies were 0.9%±1.3%, 0.5%±0.8%, and 0.9%±0.7% for M3D, CC, and CR, respectively. Comparisons did not show statistical relevance.

#### A.3 Validation with real VMAT patient plans

Point‐dose measurements taken for each treatment and comparisons with M3D and COMPASS results are shown in Fig. 2. Mean discrepancies were 0.1%±1.0%, 0.5%±1.2%, and 1.2%±0.9% for M3D, CC, and CR, respectively. The best results were found for M3D (p<0.01 for comparisons with CR). There was no statistically significant difference for other comparisons.

Mean gamma passing rates for coronal and sagittal dose planes measured with the MatriXX+MultiCube set compared with those extracted from both systems are shown in Table 4. Mean values were better than 87.0% and 97.9% for the 2%/2 mm and 3%/3 mm criteria, respectively. Differences in mean gamma passing rates between both systems were not statistically significant (lower p‐value was 0.06 for comparison between M3D and CR in the coronal plane with the 3%/3 mm criterion).

**Table 4:** Gamma analysis (2%/2 mm and 3%/3 mm, global normalization to maximum with a low‐dose threshold at 10% of global maximum) for 12 real cases: 2 head and neck, 2 lung, 2 gastric, and 6 pelvic (high‐, intermediate‐, and low‐risk prostate) treatment plans. Comparisons were performed for coronal and sagittal dose planes measured with MatriXX + MultiCube set and those extracted by Mobius3D (M3D), COMPASS dose calculation (CC), and reconstruction (CR)

	*Gamma Passing Rates (%)*										
	*Gamma* 2%/2 mm						*Gamma* 3%/3 mm					
	*Coronal*			*Sagittal*			*Coronal*			*Sagittal*		
	*M3D*	*CC*	*CR*	*M3D*	*CC*	*CR*	*M3D*	*CC*	*CR*	*M3D*	*CC*	*CR*
H&N1	90.3	78.7	79.2	83.6	84.1	80.1	97.8	95.6	96.3	98.8	97.5	98.9
H&N2	87.7	87.9	80.5	94.1	92.4	94.4	97.8	98.8	97.0	99.6	99.5	99.6
Lung1	82.8	82.4	81.9	82.3	80.2	83.7	99.4	98.0	97.5	96.1	97.8	97.3
Lung2	89.3	91.0	87.3	86.4	86.3	89.0	99.4	98.9	98.4	99.0	98.3	99.0
Gastr1	88.0	88.6	89.0	93.9	91.0	94.1	99.2	99.3	98.8	99.9	99.3	99.6
Gastr2	84.7	82.8	81.2	88.1	86.3	85.4	98.9	97.8	97.2	99.6	98.6	98.4
HRP1	97.7	81.1	83.8	95.0	89.7	95.3	100	95.3	95.4	100	99.1	99.5
HRP2	88.1	86.3	88.0	91.0	90.2	96.8	98.4	98.7	98.0	99.3	99.3	99.6
IRP1	91.3	92.2	91.0	88.6	90.4	90.1	99.5	99.6	99.5	99.2	99.7	99.8
IRP2	92.6	97.8	92.6	94.8	93.1	91.7	99.2	99.9	99.5	99.6	98.4	99.8
LRP1	94.8	94.8	94.1	91.2	94.4	95.9	100	99.5	99.9	99.4	99.5	99.7
LRP2	86.6	94.1	95.3	92.1	94.9	91.2	98.3	99.6	99.5	99.3	99.6	99.5
Mean	89.5±	88.1±	87.0±	90.1±	89.4±	90.6±	99.0±	98.4±	98.1±	99.1±	98.9±	99.2±
values	4.2	6.0	5.6	4.3	4.4	5.3	0.7	1.5	1.4	1.0	0.7	0.7

M3D=Mobius3D; CC=COMPASS dose calculation; CR=COMPASS dose reconstruction; H&N=head and neck; Gastr=gastric; HRP=high‐risk prostate; IRP=intermediate‐risk prostate; LRP=low‐risk prostate.

### B. Clinical implementation of the systems

#### B.1 Clinical implementation tests with static square fields and dynamic TG‐119 test plans

Differences between TPS, dose calculation, and reconstruction for dosimetric parameters analyzed for each structure set are shown in Table 5. Larger differences were found for high‐dose regions ($D_{99}$ in H&N, $D_{99}$ in superior and inferior volumes for MultiTarget) and for parotid glands in H&N. M3D results were better than COMPASS results for $D_{99}$ and $D_{10}$ in MultiTarget inferior volume (p=0.04 in both cases) and for H&N cord volume (p=0.02). CC results were better than M3D results for $D_{10}$ in MultiTarget superior volume (p=0.02) and right parotid gland (p=0.05) and better than CR results for $D_{20}$ in H&N (p<0.01). The remaining differences were not statistically significant. The best and the worst mean values for all parameters were observed for CC and CR, respectively. Comparisons of mean values for all TG‐119 parameters did not show statistical relevance.

**Table 5:** Comparisons of dosimetric parameters for test cases suggested by AAPM TG‐119 report. Data presented correspond to mean values for anterior, lateral, box, and VMAT fields

		*Mean Differences (%)*			*p‐values*		
		*M3D*	*CC*	*CR*	*M3D vs. CC*	*M3D vs. CR*	*CC vs. CR*
*Multitarget*						
Central	$D_{99}$	−1.0±4.3	−2.6±1.1	−0.7±5.4	0.459	0.852	0.448
	$D_{10}$	−0.5±0.5	−0.1±0.7	−0.4±0.6	0.197	0.526	0.149
Superior	$D_{99}$	2.1±7.1	5.5±5.9	1.9±3.3	0.618	0.964	0.140
	$D_{10}$	−1.3±1.1	−0.3±0.8	−2.0±1.1	0.016	0.547	0.164
Inferior	$D_{99}$	0.0±3.5	6.9±5.6	8.2±6.2	0.210	0.044	0.791
	$D_{10}$	0.1±3.3	1.2±3.3	2.0±5.8	0.038	0.250	0.558
*Mock Prostate*							
Prostate	$D_{95}$	−0.4±1.8	−1.7±0.8	−1.3±2.6	0.204	0.379	0.701
	$D_{5}$	−0.1±0.2	0.2±0.7	0.0±0.5	0.507	0.694	0.227
Rectum	$D_{30}$	−0.4±3.3	−0.4±0.6	0.9±3.9	0.995	0.175	0.485
	$D_{10}$	−0.7±1.8	−0.2±0.4	0.4±1.5	0.719	0.090	0.542
Bladder	$D_{30}$	−0.2±2.0	−0.4±0.8	−2.3±1.9	0.788	0.361	0.235
	$D_{10}$	0.2±2.2	0.0±0.4	−1.2±0.9	0.840	0.428	0.136
*Mock H&N*							
PTV	$D_{90}$	0.0±1.3	−0.6±1.0	−0.5±1.5	0.424	0.508	0.777
	$D_{99}$	9.2±13	1.2±2.9	14±22	0.214	0.400	0.286
	$D_{20}$	−0.6±0.8	−0.4±0.1	−0.8±0.0	0.702	0.621	0.005
Cord	$D_{\text{max}}$	0.0±1.3	0.5±2.8	1.1±1.5	0.564	0.015	0.525
R Parot	$D_{50}$	7.1±5.4	−0.1±2.1	1.6±5.6	0.048	0.132	0.401
L Parot	$D_{50}$	3.7±6.9	0.4±1.6	−0.1±1.5	0.355	0.337	0.371
*C‐Shape*							
PTV	$D_{95}$	0.3±3.3	−0.9±1.3	0.4±4.9	0.310	0.903	0.506
	$D_{10}$	0.2±1.6	0.0±0.2	0.4±1.2	0.819	0.579	0.608
Core	$D_{10}$	−1.4±1.0	−0.3±1.3	0.0±1.9	0.225	0.332	0.660
		*Mean differences for all parameters (%)*			*p‐values for all parameters*		
		0.8±4.2	0.4±2.3	1.0±5.8	0.55	0.69	0.34

M3D=Mobius3D; CC=COMPASS dose calculation; CR=COMPASS dose reconstruction; parot=parotid glands.

#### B.2 Clinical implementation with real VMAT patient plans

Values for the dosimetric parameters previously described are shown in Table 6. For all the parameters, mean values were 0.0%±2.3%, 0.6%±1.1%, and −0.0%±1.6% for M3D, CC, and CR, respectively. Difference was statistically significant for comparisons between both COMPASS results (p=0.01). Differences between M3D and COMPASS were not statistically significant.

Mean gamma passing rates for all structures are shown in Table 7 for three different criteria. For the local gamma tolerance, lower mean values were found for CR applied to H&N and lung treatments. For the 2%/2 mm global gamma tolerance, lower mean values were found for M3D and CR applied to gastric and high‐risk prostate treatments, respectively. CC results were better than M3D results for the 2%/2 mm global gamma criterion and better than CR results for both the 2%/2 mm and 3%/3 mm global gamma criteria (p<0.01 in all cases). The remaining differences were not statistically significant.

Gamma passing rates for the entire anatomical volume were extracted for the 12 real plans, with the previous local and global criteria. Results are shown in Table 8. Low passing rates were observed while analyzing the total volume. In order to clarify these values, 3D gamma distributions for a CC lung and CR high‐0risk prostate cases are shown in 3, 4, respectively, with gamma criteria ranging from 2%/2 mm local to 3%/3 mm global tolerance. The 2%/2 mm local gamma test highlighted gamma failing points, mainly located in low‐dose regions, where a local test could be more sensitive. The gamma passing rates observed for ORs were lower than those observed for PTVs for local gamma tests due to this effect. Relaxing gamma criteria from local to global 2%/2 mm tolerance resulted in a reduction of failing points in low‐dose regions, showing problematic areas in PTVs and surrounding regions (total passing rates with the 2%/2 mm global gamma criterion were better than 92%, excluding the second gastric treatment for M3D [81.8%]). The last step to 3%/3 mm led to passing rates better than 97.6% in all cases.

**Table 6:** Differences for dosimetric parameters (target volumes and normal tissues) obtained for 12 real VMAT plans: 2 head and neck, 2 lung, 2 gastric, and 6 prostate plans (high‐, intermediate‐, and low‐risk)

		*Differences (%)*								*Differences (%)*					
		*M3D*		*CC*		*CR*				*M3D*		*CC*		*CR*	
		*P 1*	*P 2*	*P 1*	*P 2*	*P 1*	*P 2*			*P 1*	*P 2*	*P 1*	*P 2*	*P 1*	*P 2*
*H&N*								*HR Prostate*							
PTV	$D_{98}$	7.1	5.2	−0.7	0.3	−2.8	7.3	PTV Pr	$D_{98}$	0.9	2.3	0.9	1.4	−1.1	−0.1
	$D_{2}$	0.7	−0.7	2.0	−0.5	−0.5	0.5		$D_{2}$	−0.7	0.5	2.9	−0.1	−3.1	−1.0
	$D_{50}$	0.1	0.3	0.0	−1.1	−2.2	−0.2		$D_{50}$	0.0	1.0	−0.4	0.2	−2.6	−1.6
	$D_{m}$	1.0	0.7	0.2	−0.7	−2.0	0.5		$D_{m}$	−0.1	1.1	−0.6	0.3	−2.5	−1.4
Cord	$D_{\text{max}}$	−1.6	−2.7	−5.0	−0.3	−3.1	4.6	PTV SV	$D_{98}$	−0.2	1.7	−0.8	0.4	0.2	3.1
R Parot	$D_{m}$	−5.9	−7.3	1.9	0.5	−1.9	0.9		$D_{2}$	0.3	1.4	−0.6	1.1	−2.6	−0.6
L Parot	$D_{m}$	−3.2	−8.7	0.1	0.4	−1.4	5.2		$D_{50}$	0.9	0.7	−0.3	0.3	−0.4	−1.0
									$D_{m}$	0.5	1.1	−0.3	0.6	−1.2	−0.2
*Lung*								PTV PLN	$D_{98}$	−0,8	0.2	2.0	1.5	−1.9	−2.1
PTV	$D_{98}$	4.2	2.8	0.5	0.6	0.3	0.1		$D_{2}$	0.7	0.2	−1.0	−0.8	−2.9	−2.2
	$D_{2}$	−1.6	−2.1	−1.2	−1.2	−1.9	−1.8		$D_{50}$	−2.1	−0.9	−0.5	−0.1	−2.7	−1.8
	$D_{50}$	0.3	−0.9	−1.0	−0.6	−1.9	−1.6		$D_{m}$	−1.7	−0.8	−0.4	0.0	−2.5	−1.8
	$D_{m}$	0.5	−0.6	−0.9	−0.6	−1.7	−1.5	Rectum	$V_{50}$	1.0	3.7	0.7	1.0	2.4	3.8
Cord	$D_{\text{max}}$	−6.8	3.7	−9.8	0.2	−5.0	5.1		$D_{60}$	1.5	3.0	0.5	0.9	1.7	2.9
Heart	$V_{46}$	0.1	0.2	0.1	0.0	−0.3	−0.5		$V_{65}$	1.5	2.7	0.3	0.6	2.1	2.8
	$D_{m}$	−12	−0.6	2.7	−0.4	−4.0	−2.5		$V_{70}$	1.6	2.7	0.4	0.6	1.1	2.3
R Lung	$V_{20}$	0.5	0.3	−0.2	0.6	−0.6	−1.0		$V_{75}$	1.5	2.6	0.0	0.6	1.2	2.0
	$D_{m}$	0.1	0.2	2.4	1.8	0.7	−3.0		$D_{m}$	0.4	2.9	0.8	1.4	1.1	3.9
L Lung	$V_{20}$	0.6	0.3	0.0	0.0	−0.1	−0.1	Bladder	$V_{65}$	0.7	1.3	0.4	0.9	−2.0	−2.7
	$D_{m}$	0.5	−0.8	1.3	1.7	−0.4	0.5		$V_{70}$	0.7	1.5	0.5	1.3	−1.9	−2.6
									$V_{75}$	0.5	1.3	0.8	1.4	−2.0	−2.4
*Gastric*									$V_{80}$	0,0	0.5	0.2	0.3	−3.1	−1.8
PTV	$D_{98}$	−1.0	0.5	0.5	1.1	−0.9	2.7		$D_{m}$	−1.1	0.3	0.1	0.8	−3.2	−2.7
	$D_{2}$	−0.6	−2.0	0.4	−1.7	−1.0	−1.2	R Fem	$D_{\text{max}}$	2.8	5.1	2.3	4.4	−0.4	2.7
	$D_{50}$	−0.6	−2.1	0.4	−0.7	−0.6	−1.0	L Fem	$D_{\text{max}}$	2.2	4.0	3.0	3.5	−1.6	0.7
	$D_{m}$	−0.6	−1.9	0.3	−0.6	−0.7	−0.7								
Cord	$D_{\text{max}}$	−0.9	−1.0	−0.2	−1.3	−0.3	1.5								
Liver	$V_{30}$	0.2	−0.4	0.4	0.4	0.4	0.0								
	$D_{m}$	−2.1	−1.3	1.9	0.9	1.1	0.5								
R Kidney	$V_{18}$	0.1	−1.0	0.8	0.9	1.1	4.2								
	$D_{m}$	−6.0	−2.7	4.5	1.4	3.6	5.1	*IR Prostate*						
L Kidney	$V_{18}$	−0.6	−0.3	0.8	0.8	−0.6	1.6	PTV Pr	$D_{98}$	3.4	4.3	0.6	1.1	1.5	2.6
	$D_{m}$	−5.5	−1.6	3.6	2.0	−0.3	4.2		$D_{2}$	1.1	1.2	−0.2	−0.2	−0.6	−0.7
									$D_{50}$	1.3	1.1	0.2	0.2	−0.5	−0.7
*LR Prostate*									$D_{m}$	1.6	1.8	0.0	0.3	−0.6	−0.5
PTV Pr	$D_{98}$	2.4	3.1	0.4	0.7	1.6	1.9	PTV SV	$D_{98}$	0.9	1.1	−0.7	0.1	1.0	0.7
	$D_{2}$	0.8	−0.1	0.1	−0.4	−0.6	−0.8		$D_{2}$	1.0	1.0	0.0	0.1	−0.6	−1.4
	$D_{50}$	0.4	0.1	0.2	−0.1	−0.7	−0.8		$D_{50}$	0.9	1.3	0.3	0.7	0.7	0.1
	$D_{m}$	0.7	0.4	0.2	0.0	−0.5	−0.6		$D_{m}$	1.1	−7.2	0.2	0.0	0.4	−0.6
Rectum	$V_{50}$	1.1	1.4	0.4	0.4	2.2	2.1	Rectum	$V_{50}$	2.3	1.8	0.9	0.9	3.8	3.3
	$V_{60}$	1.0	1.8	0.5	0.6	2.4	3.4		$V_{60}$	2.0	2.2	1.0	0.6	2.1	2.3
	$V_{65}$	0.9	1.9	0.1	0.1	1.6	1.5		$V_{65}$	2.0	1.9	0.4	0.4	2.2	2.6
	$V_{70}$	1.0	1.6	0.3	0.2	0.9	2.6		$V_{70}$	2.0	2.2	0.7	0.4	2.2	1.8
	$V_{75}$	1.2	2.0	0.3	1.2	1.4	2.7		$V_{75}$	2.0	2.0	0.4	0.7	1.3	2.0
	$D_{m}$	−0.1	−0.3	2.3	2.0	5.7	7.0		$D_{m}$	2.4	1.2	2.1	2.3	6.4	4.5
Bladder	$V_{65}$	0.4	1.0	0.1	0.6	−0.5	−1.9	Bladder	$V_{65}$	1.0	1.2	0.5	0.4	−1.4	−1.9
	$V_{70}$	0.3	0.8	0.1	0.8	−0.5	−1.7		$V_{70}$	0.9	1.3	0.4	0.5	−1.4	−2.0
	$V_{75}$	0.1	0.8	0.1	0.5	−0.4	−2.3		$V_{75}$	0.9	1.4	0.4	0.5	−1.3	−2.0
	$V_{80}$	0.0	−0.9	0.0	0.0	0.0	−3.5		$V_{80}$	0.3	0.6	0.0	0.2	−0.6	−1.8
	$D_{m}$	−5.2	−1.3	4.9	2.5	−2.4	−3.4		$D_{m}$	−1.2	1.1	2.4	2.3	−3.3	−3.7
R Fem	$D_{\text{max}}$	−0.3	0.0	0.6	2.2	2.2	1.5	R Fem	$D_{\text{max}}$	0.0	0.9	2.1	2.0	2.6	2.8
L Fem	$D_{\text{max}}$	0.7	−0.5	1.9	1.3	0.6	0.1	L Fem	$D_{\text{max}}$	1.9	1.0	2.3	2.6	2.0	3.0

M3D=Mobius3D; CC=COMPASS dose calculation; CR=COMPASS dose reconstruction; P=patient; H&N=head and neck; parot=parotid glands; Pr=prostate; SV=seminal vesicles; PLN=pelvic lymph nodes; Fem=femoral heads.

**Table 7:** Mean local gamma passing rates (2%/2 mm, local normalization) for the COMPASS system and mean global gamma passing rates (2%/2 mm and 3%/3 mm, global normalization to maximum with a low‐dose threshold at 10% of global maximum) for the COMPASS and Mobius3D systems. Results were extracted for all structures and locations of the 12 real VMAT plans

	*Gamma Passing Rates (%)*					
	*H&N*	*Lung*	*Gastric*	*HRP*	*IRP*	*LRP*
*Local Gamma* 2%/2 mm						
CC	83±14	66±23	72±22	84±13	87±13	82±17
CR	65±13	65±14	78±14	75±20	83±14	83±14
*Global Gamma* 2%/2 mm						
M3D	93±11	91±13	88±17	91.0±9.6	94.6±5.9	95.4±4.7
CC	95.8±6.3	98.0±4.3	98.3±4.6	96.7±3.1	99.8±0.2	99.3±0.9
CR	91±15	92±13	94.7±7.0	86±18	94.8±4.6	95.2±6.1
*Global Gamma* 3%/3 mm						
M3D	98.8±2.3	97.8±4.8	97.5±7.8	99.4±1.3	99.3±1.5	99.9±0.2
CC	98.8±2.6	99.7±0.7	99.7±1.1	99.7±0.6	100±0.0	100±0.1
CR	95.8±7.6	96.0±9.3	93±16	96.4±5.3	97.6±5.0	97.9±3.8

M3D=Mobius3D; CC=COMPASS dose calculation; CR=COMPASS dose reconstruction; H&N=head and neck; HRP=high‐risk prostate; IRP=intermediate‐risk prostate; LRP=low‐risk prostate.

**Table 8:** Local gamma passing rates (2%/2 mm, local normalization) for the COMPASS system, and mean global gamma passing rates (2%/2 mm and 3%/3 mm, global normalization to maximum with a low‐dose threshold at 10% of global maximum) for the COMPASS and Mobius3D systems. Results were extracted for the entire volume of the 12 real VMAT plans

	*Total Gamma Passing Rates (%)*											
	*H&N*		*Lung*		*Gastric*		*HRP*		*IRP*		*LRP*	
	*Pat. 1*	*Pat. 2*	*Pat. 1*	*Pat. 2*	*Pat. 1*	*Pat. 2*	*Pat. 1*	*Pat. 2*	*Pat. 1*	*Pat. 2*	*Pat. 1*	*Pat. 2*
*Local Gamma* 2%/2 mm												
CC	54.7	36.3	42.1	45.0	35.4	61.0	79.9	68.2	39.4	41.4	44.6	39.5
CR	29.0	18.4	36.0	45.0	46.2	57.1	51.4	56.2	42.6	39.3	43.8	43.2
*Global Gamma* 2%/2 mm												
M3D	92.7	99.1	97.7	97.8	96.5	81.8	94.6	95.7	98.8	98.7	98.4	99.0
CC	98.5	99.9	99.7	99.8	99.8	97.5	97.8	98.4	99.9	100.0	99.8	99.9
CR	93.8	99.3	98.8	97.8	98.1	95.1	93.2	96.9	98.9	96.6	97.6	98.4
*Global Gamma* 3%/3 mm												
M3D	99.6	99.9	99.7	99.9	99.9	97.6	99.7	100.0	100.0	99.9	99.9	100.0
CC	99.8	100.0	100.0	100.0	100.0	99.6	99.8	99.8	100.0	100.0	100.0	100.0
CR	98.4	99.8	99.8	99.7	99.9	99.3	99.2	99.7	99.9	99.0	99.5	99.8

M3D=Mobius3D; CC=COMPASS dose calculation; CR=COMPASS dose reconstruction; H&N=head and neck; HRP=high‐risk prostate; IRP=intermediate‐risk prostate; LRP=low‐risk prostate.

**Figure 3:**
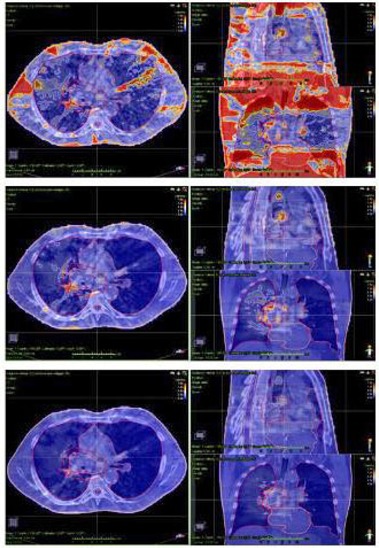
3D gamma distributions (axial, coronal, and sagittal) for a COMPASS calculated lung treatment with three different criteria: (a) 2%/2 mm local, (b) 2%/2 mm global, and (c) 3%/3 mm global. Failing points for local gamma test were mainly located in low‐dose regions, where the local tolerance could be more sensitive. Relaxing gamma criteria from local to global tolerance resulted in a reduction of failing points in low‐dose regions, showing problematic areas in PTVs and surrounding areas.

**Figure 4:**
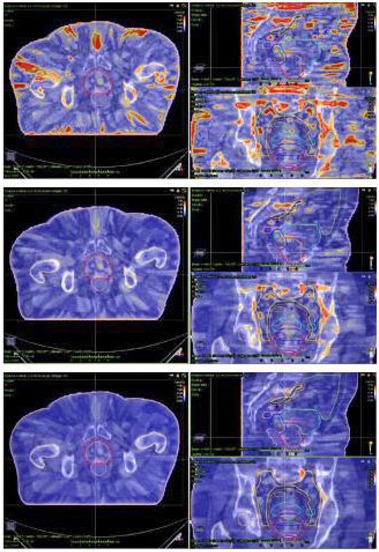
3D gamma distributions (axial, coronal, and sagittal) for a COMPASS reconstructed high‐risk prostate treatment with three different criteria: (a) 2%/2 mm local, (b) 2%/2 mm global, and (c) 3%/3 mm global. Failing points for local gamma test were mainly located in peripheral low‐dose regions, where the local tolerance could be more sensitive. Relaxing gamma criteria from local to global resulted in a reduction of failing points in low‐dose regions. Good passing rates were kept for PTV in both local and global tests.

## IV. DISCUSSION

### A. Remarks and limitations of the present study

#### A.1 MatriXX spatial resolution

Geometrical resolution of 2D detector arrays is limited due to the size of each single detector. Strictly, Mobius3D‐ and COMPASS‐calculated values in planar analysis should be convolved with the detector response function and then compared to MatriXX measurements.^(47)^ This problem, described as a limitation of the present study, is not observed in measurement‐based dose reconstruction performed by COMPASS because the system inherently applies this correction.

#### A.2 Gamma passing rate metric in this study: application of tighter tolerances in gamma analysis

A 3%/3 mm gamma passing rate metric is commonly used in QA tasks.^(21)^ However, common metrics may reduce the sensitivity of systems involved in patient‐specific QA processes.^15^, ^25^, ^26^, ^48^, ^49^, ^50^ A recent study by Nelms et al.^(50)^ suggested performing a more stringent gamma analysis, restricting traditional tolerances. Validation of both systems, comparing planar dose distributions by means of gamma analysis, involved 2%/2 mm and 3%/3 mm global gamma criteria. Clinical implementation of the two systems made use of 2%/2 mm and 3%/3 mm global tolerances in volumetric dose comparisons and introduced a 2%/2 mm local gamma test for COMPASS results. The implementation of previous tolerances (local/global) in the analysis of each case (planar/volumetric) was a limitation of this study. OmniPro I'mRT was used to perform 2D gamma analysis (validation of the systems). Mobius3D was used to perform 3D gamma analysis (clinical implementation of the systems). Global gamma normalization is the only tolerance available in the previous solutions. COMPASS, however, is able to perform local and global gamma analysis in volumetric comparisons.

#### A.3 Remarks about evaluation and comparison of Mobius3D and COMPASS

Several authors have presented commissioning studies for 3D pretreatment verification systems.^27^, ^51^, ^52^, ^53^ A comparison of Mobius3D with other solutions has not previously been evaluated in the literature. TG‐119‐based comparisons are powerful tools to evaluate the performance of IMRT and VMAT TPSs^(54)^ and can also be implemented to evaluate DVH‐based QA systems.

Validation of the systems was performed by means of comparisons with measurements taken with external devices and usual metrics (point‐dose comparisons and planar gamma analysis). Clinical implementation was also performed in terms of TG‐119 and real plan comparisons with usual (planar and volumetric gamma analysis) and DVH‐based metrics.

An additional limitation is described for this study. Mobius3D has an independent tool to predict dose on patient anatomy, called MobiusFX (Mobius Medical Systems). This software reconstructs the dose from delivery (log‐file) information. At the time of the present study, this tool is not available in our institution. Future work should focus on validation and clinical implementation of the MobiusFX tool, as a counterpart of the COMPASS dose reconstruction scheme.

### B. Validation of the systems

#### B.1 Comparison with reference calibration

Comparisons of absolute dose values obtained with M3D and COMPASS were comparable with those from previous studies.^27^, ^51^, ^55^ Mean differences for M3D at 10−20 cm of depth were −2.7%±0.2%, compared with −1.3%±0.2% for CC and −1.4%±0.2% for CR. The mean gamma passing rate for M3D (2%/2 mm, local normalization) is 68%±28%. These discrepancies can probably be improved by adjusting the reference data in M3D. The largest differences observed in profile comparisons were located in the penumbra region. The steep dose gradient present in this area contributes to increasing the discrepancies, as can be observed from gamma passing rates (mean values were better than 98% for the 2%/2 mm criterion with local normalization). However, profile comparisons resulted in good agreement between both systems and reference data. As a conclusion, it can be assumed that CC results were better than M3D and CR results in most situations. Passing rates were above the TG‐119 action level of 90% for individual field dose gamma analysis^(21)^ for PDDs and profiles, excluding some fields evaluated with the 2%/2 mm tolerance (M3D PDDs for 2×2, 3×3, 4×4, and $10\times 10\;{\text{cm}}^{2}$ fields).

#### B.2 Validation tests with static square fields and dynamic TG‐119 test plans

Mean discrepancies were better than 1.0%. Results for static fields were comparable to those obtained in the previous section. For dynamic TG‐119 plans, values were comparable to those extracted with real VMAT plans.

#### B.3 Validation with real VMAT patient plans

Results for comparisons with ion chamber measurements were comparable or better than those found in the literature.^27^, ^51^, ^55^ Values for M3D were slightly better than those from CC and CR in point‐dose comparisons. Planar dose comparisons led to similar results for the two systems, and they were better for sagittal dose planes. Passing rates were above the TG‐119 action level of 88% for composite dose gamma analysis,^(21)^ excluding mean values of coronal planes for CR evaluated with the 2%/2 mm tolerance.

### C. Clinical implementation of the systems

#### C.1 Clinical implementation tests with static square fields and dynamic TG‐119 test plans

Results for TG‐119 test suite comparisons were consistent with previous studies. Dose‐volume discrepancies were also comparable with actual clinical plans validated by other authors.^27^, ^50^, ^55^ The observed differences could be reduced by improving the beam model for the Mobius3D and COMPASS systems. In the dose reconstruction process, the spatial resolution of the MatriXX detector could be a problem for detecting hot/cold spots in highly modulated fields and might contribute to obtaining worse results.

#### C.2 Clinical implementation with real VMAT patient plans

Dose‐volume comparisons were comparable with other studies.^27^, ^50^, ^55^ Differences between both COMPASS results were significant, but M3D results compared with those from the COMPASS system led to p‐values higher than 0.05. These discrepancies could be improved with the same solutions reported in the previous sections. These results and those described in the previous section were in contrast with statistical analysis of relative dose distributions performed in the first section. Results for both systems were comparable in terms of dose‐volume parameters. Gamma passing rates with global normalization were above the TG‐119 action level of 88% for composite dose gamma analysis.^(21)^

## V. CONCLUSIONS

The Mobius3D and COMPASS systems have been tested with quality assurance, static, and dynamic plans, resulting in good agreement with validation measurements. In addition, tests performed to evaluate the clinical implementation of both systems by means of comparisons with TPS calculations are in good agreement, according to dosimetric benchmarks. The two systems can be clinically implemented with no significant differences between them.

## Supporting information

Supplementary Material

Supplementary Material

Supplementary Material
