# Supplementary figures and images for: Dosimetric validation and clinical implementation of two 3D dose verification systems for quality assurance in volumetric‐modulated arc therapy techniques
Source: J Appl Clin Med Phys. 2015 Mar 8;16(2):198–217. doi: 10.1120/jacmp.v16i2.5190 (PMC5690088; doi:10.1120/jacmp.v16i2.5190)

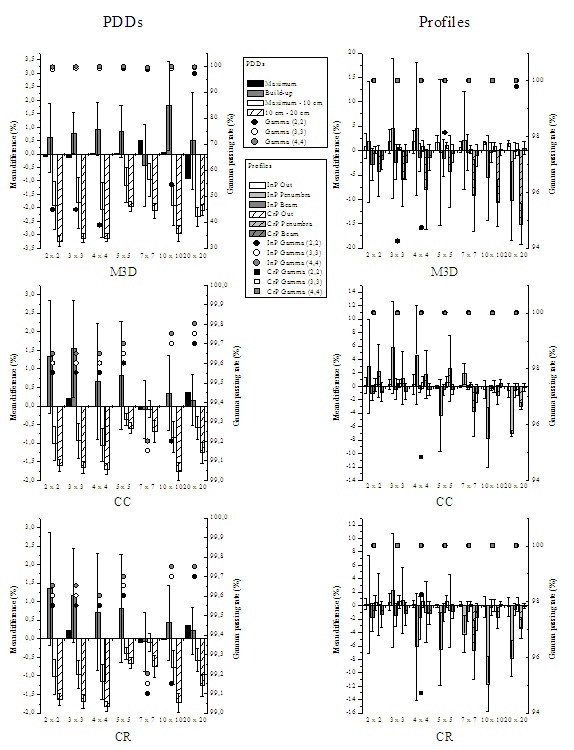

Supplement: Supplementary file 1 — Supplementary Material [file ACM2-16-198-s001.jpg]

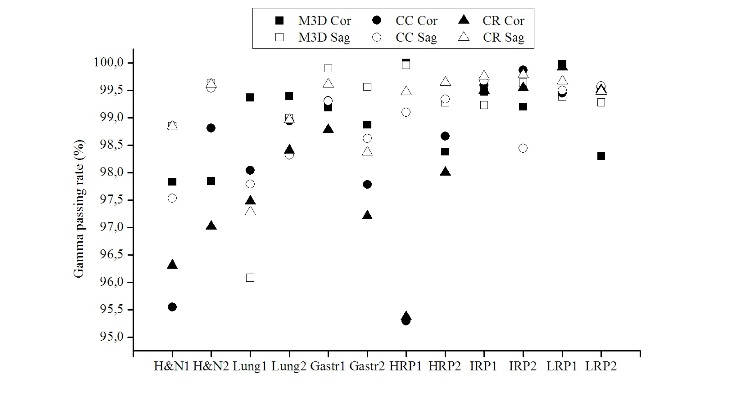

Supplement: Supplementary file 2 — Supplementary Material [file ACM2-16-198-s002.jpg]

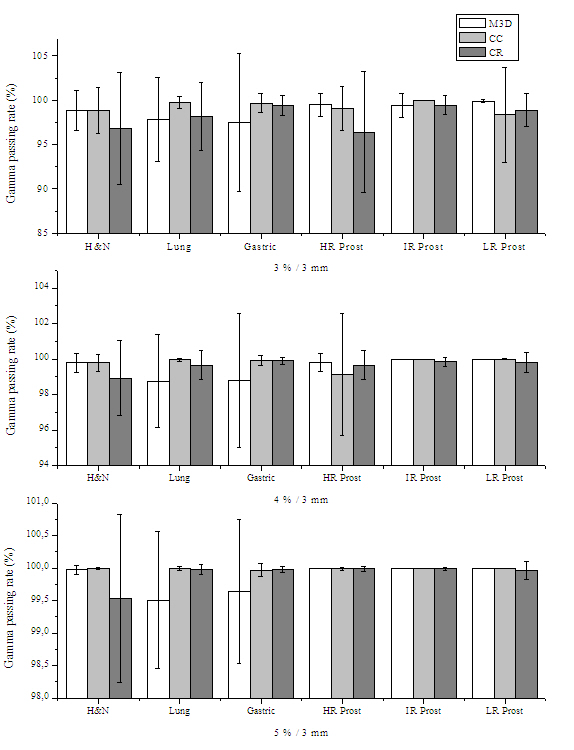

Supplement: Supplementary file 3 — Supplementary Material [file ACM2-16-198-s003.jpg]
